# Patient Experiences With a Symptom Monitoring and Self-Referral Program for Specialty Palliative Care After Lung Cancer Diagnosis: Qualitative Study

**DOI:** 10.2196/95948

**Published:** 2026-09-24

**Authors:** Julia L Agne, Alice A Gaughan, Laura J Rush, Jennifer L Eramo, Anneliese Sinclair, Madison M Grogan, Jessica S Merlin, Jack Stevens, Robert M Cronin, Tasleem J Padamsee, Carolyn J Presley, Ann Scheck McAlearney

**Affiliations:** 1 Section of Palliative and Pain Research (SPARC) Division of Palliative Medicine The Ohio State University Wexner Medical Center Columbus, OH United States; 2 Center for Research Excellence in Supportive Care (CREST) The Ohio State University Comprehensive Cancer Center – Arthur G. James Cancer Hospital and Richard J. Solove Research Institute Columbus, OH United States; 3 Center for the Advancement of Team Science, Analytics, and Systems Thinking in Health Services and Implementation Science Research (CATALYST) College of Medicine The Ohio State University Columbus, OH United States; 4 College of Medicine The Ohio State University Columbus, OH United States; 5 Division of Medical Oncology Department of Internal Medicine The Ohio State University Comprehensive Cancer Center – Arthur G. James Cancer Hospital and Richard J. Solove Research Institute Columbus, OH United States; 6 Department of Pediatrics The Ohio State University Columbus, OH United States; 7 Nationwide Children's Hospital Columbus, OH United States; 8 Department of Internal Medicine The Ohio State University Wexner Medical Center Columbus, OH United States; 9 Division of Health Services Management and Policy College of Public Health The Ohio State University Columbus, OH United States; 10 Department of Family and Community Medicine The Ohio State University Columbus, OH United States

**Keywords:** cancer, mHealth, mobile health, palliative care, qualitative, remote symptom monitoring, self-referral, symptom monitoring program

## Abstract

**Background:**

Palliative care improves patients’ health-related quality of life (HRQoL) by delivering specialty-level management for cancer symptoms. Digital symptom monitoring programs (SMPs) improve patients’ HRQoL through early symptom identification during cancer care. Through complementary approaches, SMPs and palliative care align with symptom management theory by linking the patient-reported symptom experience with clinician recommendations for symptom management to improve health outcomes for patients with serious illness. While both SMPs and palliative care reduce health care use and improve survival through detection and treatment of cancer symptoms, few studies have attempted to integrate palliative care into SMPs for cancer symptom management.

**Objective:**

This study aimed to assess patients’ perspectives about the feasibility and acceptability of an SMP with a prompted palliative care self-referral option (Symptom Monitoring Program Linked to Electronic Referrals [SyMPLER]) via a mobile health (mHealth) app during lung cancer treatment.

**Methods:**

This is a single-institution qualitative study of patients newly diagnosed with lung cancer who were enrolled in a single-arm feasibility pilot of an mHealth app that facilitates on-demand reporting of cancer-related symptoms and prompts patients to self-refer to palliative care for symptom management. Interview participants were purposively sampled based on high (≥3 uses) vs low (0-2 uses) engagement with the app during the first 3 months of access to the SMP. Questions focused on program feasibility and acceptability as well as perceptions of the 5 components of the SMP intervention (introduction to palliative care, reminders, symptom reporting, palliative care self-referral, and oncology team callback). Interviews were conducted from May 2024 to May 2025 by telephone, recorded, transcribed, and analyzed using thematic analysis.

**Results:**

A total of 31 patients were interviewed; 21 had high and 10 had low app engagement. Participants were mostly White (n=29, 93.5%), female (n=21, 67.7%), and aged 60 years or older (n=19, 61.3%). In high and low engagement groups, participants voiced willingness and ability to report symptoms through the app and self-refer to palliative care if needed for symptom management. Feasibility barriers differed by group, with high-engagement participants noting forgetting to use the app for symptom reporting, while low-engagement participants cited low symptom burden, technology concerns, and time constraints as reasons for limited use of the SMP. Participants described uncertainty about when or why to request palliative care as a major barrier to self-referral.

**Conclusions:**

Participants’ perspectives on the feasibility and acceptability of SyMPLER differed by their level of engagement with the SMP app. Participants in both groups voiced acceptance of the option to self-refer to palliative care, although feasibility was limited by low uptake of palliative care self-referral. Participants suggested improvements to educational outreach, digital accessibility, customizable reminders, and recommendations for help-seeking behaviors based on symptom scoring thresholds to improve patient engagement with iterations of this SMP intervention.

## Introduction

Palliative care is specialized medical care that improves patients’ quality of life (QoL) through the early identification and treatment of symptoms arising from serious illness [1,2]. This is particularly important in lung cancer, where >90% of patients report at least 1 uncontrolled, cancer-related symptom [3]. Through an interdisciplinary approach to symptom management, palliative care improves symptom burden, health-related quality of life (HRQoL), and overall survival for patients living with advanced lung cancer [4,5]. However, oncology clinicians underestimate symptom burden and its impact on patients’ QoL, thereby limiting palliative care referrals primarily to patients and families who can clearly articulate concerns about uncontrolled symptoms [6-8].

Symptom monitoring programs (SMPs) partially fill this gap by improving patient-clinician communication about symptoms during cancer care. Informed by symptom management theory [9,10], SMPs provide a digital interface through which patients can report their symptoms using validated survey measures [11,12]. SMPs then alert oncology clinicians to uncontrolled symptoms when survey responses exceed predefined severity thresholds. Like palliative care, SMPs also improve symptom burden, HRQoL, and overall survival for patients living with advanced lung cancer [12-15]. When patients routinely participate in symptom reporting, the effectiveness of the SMP relies on oncology clinicians’ recommendations for managing uncontrolled symptoms, as symptom reporting alone, without clinician response to uncontrolled symptoms, is nonsuperior to usual care [16,17]. Although consensus guidelines strongly recommend early referrals to specialty palliative care for cancer symptom management [18,19], prior SMPs have not impacted clinician-ordered referrals to specialty palliative care, despite high patient-reported symptom burden [20,21]. Therefore, closer integration of palliative care into SMPs remains a unique opportunity to improve patients’ experiences and health outcomes through specialty-level symptom management when uncontrolled symptoms occur.

To integrate palliative care into an SMP, we developed Symptom Monitoring Program Linked to Electronic Referrals (SyMPLER), which is a multicomponent digital health intervention delivered via a patient-facing mobile health app. SyMPLER’s five main components (Figure 1) include (1) an introduction to palliative care via online and print educational materials curated by the Center for the Advancement of Palliative Care [22], (2) weekly SMS text reminders encouraging participants to report symptoms, (3) on-demand symptom reporting using a modified version of the Edmonton Symptom Assessment System (ESAS; Edmonton Symptom Assessment System–Revised Version Including Constipation and Sleep [ESAS-r-CS] [23]) via an app-based interface [24], (4) a palliative care self-referral prompted via the study app, and (5) an option to request a telephone call from their oncology team within the next 3 days to discuss their symptoms.

At SyMPLER enrollment, study coordinators provided patients with a tablet device to view online educational content about palliative care one time in the oncology clinic (component 1). Online content included a definition of palliative care (“specialized medical care for people living with a serious illness”), the embedded video “Palliative Care: YOU Are a Bridge” (1 minute, 43 seconds), and a written description of palliative care’s role in symptom management and goal-concordant care [22]. Study coordinators also provided patients with the printed handout, “*Palliative Care: What You Should Know*,” which includes 7 frequently asked questions with answers curated by the Center to Advance Palliative Care (CAPC) [25]. Patients were then registered to receive text reminders every Monday at noon (component 2) via an SMS text messaging service [26]. Finally, study coordinators assisted patients in downloading the study app on their smartphone device (Figure 2) and counseled patients to use the app as often as desired to report symptoms and request help (components 3-5) over the next 6 months. To protect patients’ privacy during symptom monitoring, SyMPLER used the MyCap app interface to ensure real-time, secure transfer of participants’ symptom survey responses to an encrypted database behind the medical center’s firewall without local storage on the participants’ smartphone devices [24].

Patients reported symptoms on the app using the ESAS-r-CS [23], a validated survey in which respondents rate the severity of 10 symptoms (ie, pain, fatigue, drowsiness, nausea, anorexia, dyspnea, depression, anxiety, constipation, and insomnia) and overall well-being on a 0 to 10 scale (0=no issue; 10=worst possible) over the last 24 hours. After completing the ESAS-r-CS, the app asked the patient if they wanted help for symptom management via a palliative care self-referral (“Do you want to see a palliative care specialist at your next oncology visit?”) and/or oncology clinician callback (“Do you want a member of the medical team to contact you within the next 3 days to discuss your symptoms?”). Upon responding to the palliative care self-referral prompt, the app provided a multioption list with free-text fields where the patient could indicate their rationale for requesting or declining palliative care. As there is no consensus on symptom survey thresholds for palliative care referral or oncology clinician callback [27,28], patients received both prompts irrespective of symptom severity reported on the ESAS-r-CS. When a patient requested palliative care referral or oncology clinician callback, the app immediately alerted the study team, who routed the request to the patient’s oncology clinicians in the electronic health record (EHR). While oncology clinicians could decline a request for palliative care referral or callback, this was not observed in the SyMPLER pilot study. The app forwarded symptom scores (ESAS-r-CS) to the palliative care or oncology teams only when a patient requested palliative care referral or oncology clinician callback, respectively (Figure 1).

We hypothesized that integration of palliative care into an SMP may overcome referral barriers [29,30] by providing patients with direct access to palliative care specialists when they wanted help for symptom management. Therefore, the primary aim of this qualitative study was to explore patients’ perspectives about the feasibility and acceptability of integrating palliative care self-referral into an SMP after a lung cancer diagnosis. Findings from this study can inform future efforts to improve the SyMPLER digital health intervention prior to testing its effectiveness and implementation on a larger scale.

**Figure 1:**
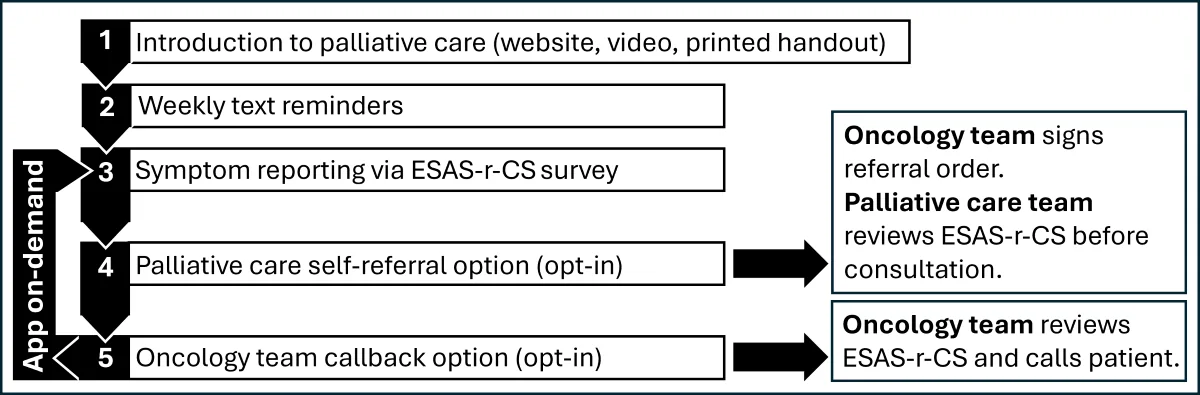
Symptom Monitoring Program Linked to Electronic Referrals (SyMPLER) intervention components in sequential order. SyMPLER participants were recruited from the Ohio State University Thoracic Oncology Clinic in Columbus, Ohio, United States. Recruitment for qualitative interviews occurred from May 2024 to May 2025. ESAS-r-CS: Edmonton Symptom Assessment System–Revised Version Including Constipation and Sleep.

**Figure 2:**
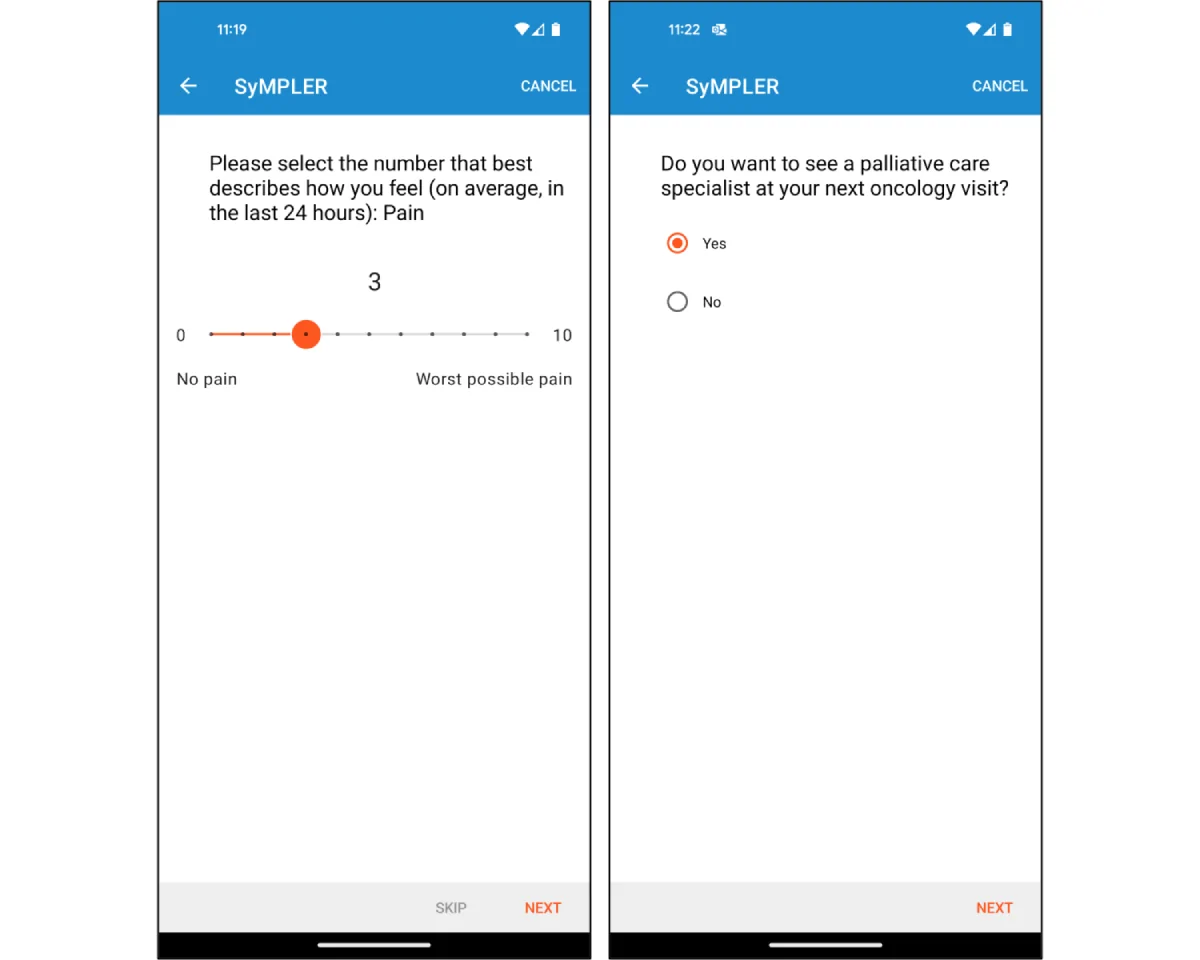
Study app interface. Screenshots of Symptom Monitoring Program Linked to Electronic Referrals (SyMPLER) in the MyCap app interface. SyMPLER participants were recruited from the Ohio State University Thoracic Oncology Clinic in Columbus, Ohio, United States. Recruitment for qualitative interviews occurred from May 2024 to May 2025.

## Methods

### Ethical Considerations

The study was conducted in accordance with the Declaration of Helsinki and was approved by the Institutional Review Board (IRB) at The Ohio State University in Columbus, Ohio, United States (2023C0182, 2023C0142). All participants provided written informed consent prior to enrollment in the SyMPLER pilot study and verbal informed consent prior to being interviewed. Research staff informed participants that they could withdraw from the study, refrain from answering any question, or stop an interview at any time without penalty. Research staff deidentified all data prior to coding and analysis. Participants received a US $25 gift card after each interview.

### Sample Selection and Recruitment

Research staff recruited interviewees from among participants enrolled in the SyMPLER pilot study (NCT06396598), a prospective single-arm feasibility study of the SyMPLER digital health intervention in patients with lung cancer. Lung cancer was chosen as the target of SyMPLER due to its high symptom burden across all cancer stages during the first 6 months after diagnosis [3]. The SyMPLER pilot study included adults (aged 18 years or older) with any stage thoracic malignancy (non–small cell lung cancer, small cell lung cancer, mesothelioma, or thymic carcinoma) who were within 12 weeks of their first outpatient oncology appointment at the Ohio State University Comprehensive Cancer Center. Inclusion criteria for the SyMPLER pilot study required participants to have access to a smartphone device, speak and read English, and not have been referred to outpatient palliative care prior to study enrollment. Of patients screened for the SyMPLER pilot study, >99% had access to a smartphone device. Research staff approached all screen-eligible patients for participation in the SyMPLER parent study at this National Cancer Institute Comprehensive Cancer Center.

After pilot study enrollment, research staff approached SyMPLER participants via email inviting them to participate in qualitative interviews upon meeting one of the following eligibility criteria: 3 instances of using the study app for symptom reporting or 3 months after enrollment in the SyMPLER pilot study. Reflective of other cancer SMPs reported in the literature [21,31,32], the SyMPLER pilot study defined feasible patient engagement a priori as an average of ≥1 app use per month. Using this definition, interview participants were purposively recruited [33] based on high (≥3 app uses) vs low (<3 app uses) engagement with symptom reporting during the first 3 months of SyMPLER enrollment. No additional eligibility criteria were required for participation in qualitative interviews. Research staff contacted potential participants up to 3 times via telephone to invite them to participate in interviews. For those willing to participate, verbal consent was obtained at the beginning of each interview. Recruitment for interviews ended when thematic saturation was achieved in both high and low engagement groups [34].

### Development of Interview Guide

The research team developed a semistructured interview guide to explore participants’ perspectives on overall feasibility and acceptability of the SyMPLER intervention, helpfulness and challenges of each component, and suggestions for improvement. The interview guide was developed using a pragmatic qualitative inquiry approach [35,36] to specifically answer the research question of whether the SyMPLER intervention, and each of its component parts, was feasible and acceptable to patients with lung cancer for symptom reporting outside the health care setting. To conceptualize this inquiry, the interview guide included questions related to the 3 domains (person, disease, and environment) and 3 dimensions (symptom experience, management, and outcomes) of symptom management theory [9,10]. The interview guide, annotated by research inquiry (feasibility, acceptability, intervention component, and suggestions for improvement) and aspects of symptom management theory (domain and dimension), is provided in Multimedia Appendix 1.

If a participant requested a palliative care referral or oncology team callback for the first time after their initial interview, research staff contacted the participant to complete a second interview about their experience requesting help through the SyMPLER app. The same interview guide was used for second interviews. Pilot testing of the interview guide was not conducted as the semistructured guide was adapted during use to refine the conversational flow and probe additional details as emerging themes arose during interviews.

### Data Collection

Participants’ contact information and demographics were collected per the SyMPLER pilot study protocol. One member of the research team (JLE) conducted telephone interviews using a semistructured interview guide (Multimedia Appendix 1) from May 2024 to May 2025. All interviews were audio recorded, transcribed verbatim, and deidentified prior to data analysis. During this qualitative study, all research team members were blinded to participants’ demographics, cancer diagnoses, and any survey responses, including ESAS-r-CS, collected during the larger SyMPLER pilot study.

### Data Analysis

Interview transcripts were coded and analyzed using both deductive and inductive thematic analysis [37]. Five members of the research team (JLA, AAG, ASM, LJR, and JLE) initially met to develop a preliminary coding dictionary based on the 8 pragmatic topics addressed in the semistructured interview guide (ie, program feasibility, acceptability, 5 intervention components, and suggestions for improvement). Coding was then completed by a primary coder (AAG) with over 10 years of qualitative coding experience using deductive, dominant thematic analysis [36,37]. The primary coder was supported by a research assistant who also participated in the coding process (AS). These 2 coders met twice weekly throughout the coding process with the senior qualitative researcher (ASM) to discuss the application of codes. Questions about application of the codes were resolved through these group discussions.

The coders (AAG and AS) and senior qualitative researcher (ASM) met twice monthly with the study’s principal investigator (JLA) to discuss ongoing findings and emergent codes characterizing participants’ perceptions of the SyMPLER intervention. These group discussions informed the refinement of code definitions and addition of emergent codes to the coding dictionary. Specifically, new codes emerged as different subthemes developed, and findings suggested the distinction between high-engagement and low-engagement participants. Findings were also discussed monthly by the broader research team, ensuring rigor and trustworthiness [38]. Saturation was reached to the extent that no additional themes were identified in the dataset [39].

The coding and analysis process was supported using ATLAS.ti (version 25.0.1; ATLAS.ti Scientific Software Development GmbH). The final codebook is provided in Multimedia Appendix 2. This work follows the Standards for Reporting Qualitative Research (SRQR; Multimedia Appendix 3; [40]).

## Results

### Overview

Research staff approached 60 participants for semistructured interviews upon meeting one of the eligibility criteria, representing 63.8% (60/94) of those enrolled in the SyMPLER pilot study. Of the 31 who consented to interviews, 21 participants had high engagement with the study app, whereas 10 participants had low engagement prior to being interviewed. Individual interviews lasted a median of 19 (IQR 14.9-24.5; range 2-36) minutes. Figure 3 provides a flow diagram of recruitment, enrollment, and participant-reported reasons for declining to be interviewed**.**

To answer the research question of whether the SyMPLER intervention and all its components are feasible and acceptable to patients with lung cancer, participant characteristics (Table 1), qualitative themes, and suggestions for improvement are presented by and compared between high-engagement and low-engagement groups. Table 2 provides representative participant quotes listed by qualitative themes and group designation.

**Figure 3:**
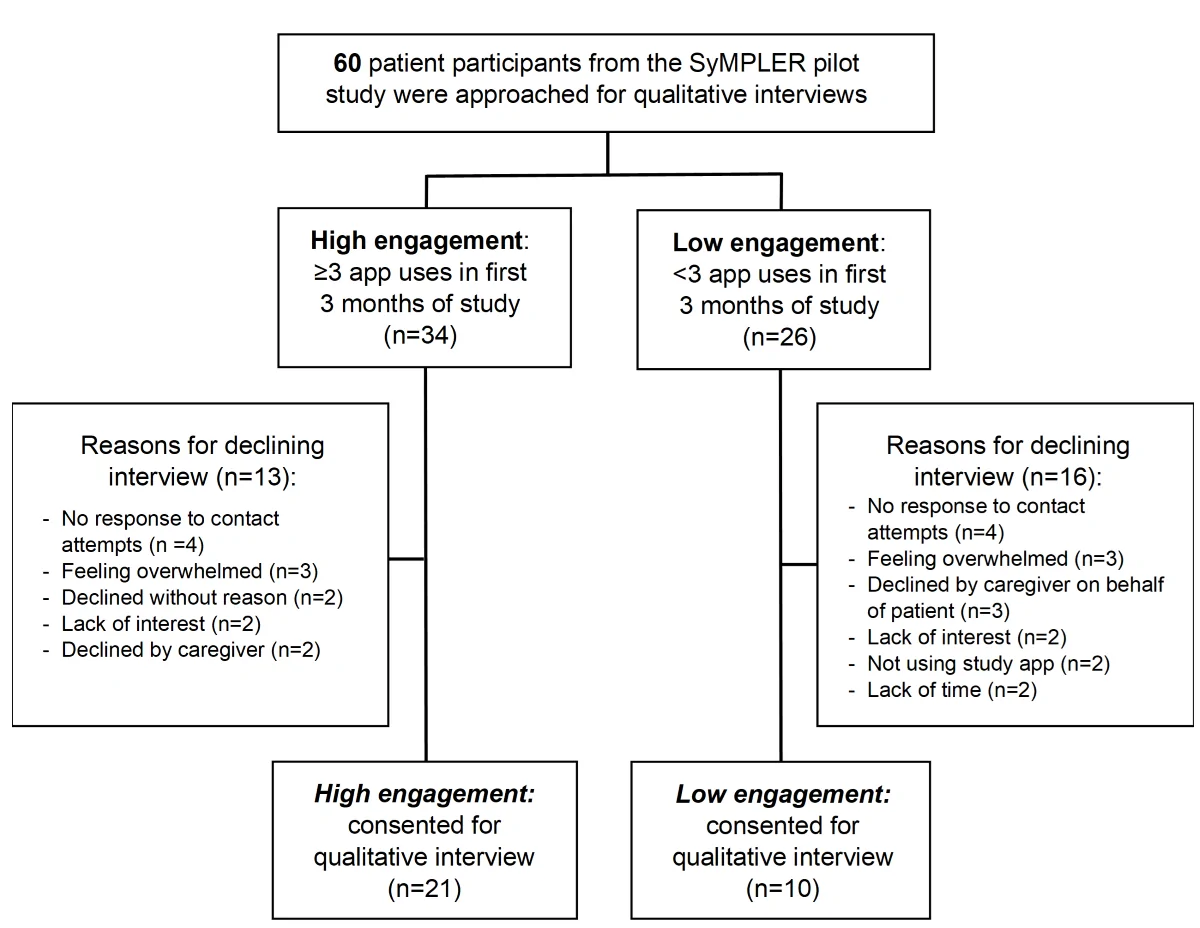
Recruitment flow diagram. Flow diagram outlining recruitment of interviewees from among patients with lung cancer also enrolled in the Symptom Monitoring Program Linked to Electronic Referrals (SyMPLER) pilot study. SyMPLER participants were recruited from the Ohio State University Thoracic Oncology Clinic in Columbus, Ohio, United States. Recruitment for qualitative interviews occurred from May 2024 to May 2025.

**Table 1:** Participant characteristics by level of engagement. Symptom Monitoring Program Linked to Electronic Referrals (SyMPLER) participants were recruited from the Ohio State University Thoracic Oncology Clinic in Columbus, Ohio, United States. Recruitment for qualitative interviews occurred from May 2024 to May 2025.

Characteristic		High engagement (n=21)	Low engagement (n=10)
Age (years), median (IQR)		60 (51-67)	64 (58-70)
Female sex, n (%)		15 (71.4)	6 (60)
Race, n (%)			
	White	19 (90.4)	10 (100)
	Black	1 (4.8)	0 (0)
	Asian	1 (4.8)	0 (0)
Marital status, n (%)			
	Married	16 (76.2)	8 (80)
	Domestic partner	0 (0)	0 (0)
	Widowed	1 (4.8)	0 (0)
	Divorced	2 (9.5)	1 (10)
	Single	2 (9.5)	1 (10)
Education, n (%)			
	High school	5 (23.8)	3 (30)
	Some college	8 (38.1)	4 (40)
	Technical school	0	2 (20)
	Undergraduate degree	2 (9.5)	0 (0)
	Advanced degree	6 (28.6)	1 (10)
Distance to clinic (miles), median (IQR)		68.00 (37.2-95.9)	19.35 (11.3-52.4)
Insurance type, n (%)			
	Medicare	6 (28.6)	4 (40)
	Medicaid	0 (0)	0 (0)
	Private	13 (61.9)	6 (60)
	Medicare + private	2 (9.5)	0 (0)
Cancer diagnosis and stage, n (%)			
	NSCLC^a^, stage 1	1 (4.8)	0 (0)
	NSCLC, stage 2	2 (9.5)	3 (30)
	NSCLC, stage 3	2 (9.5)	4 (40)
	NSCLC, stage 4	14 (66.7)	2 (20)
	SCLC^b^, extensive	2 (9.5)	0 (0)
	Large cell, stage 2	0 (0)	1 (10)

^a^NSCLC: non–small cell lung cancer.

^b^SCLC: small cell lung cancer.

**Table 2:** Representative quotes by qualitative themes and participant groups. Symptom Monitoring Program Linked to Electronic Referrals (SyMPLER) participants were recruited from the Ohio State University Thoracic Oncology Clinic in Columbus, Ohio, United States. Recruitment for qualitative interviews occurred from May 2024 to May 2025.

Theme and group		Representative quotes^a^
Feasibility		
	High	“It’s very easy. I mean very super easy to use, fast...I love the reminders every week to get in there, the text reminder. And I mean it’s again, it’s just super easy and fast. It only takes five minutes to go through and run through the questions really fast. So, I think it’s been a good experience.”‬ ‬‬‬‬‬‬‬‬‬‬‬‬‬‬‬‬‬‬‬‬‬‬(Participant 6)‬‬‬‬‬‬‬‬ “It’s super simple, it doesn’t take much time. Yeah. That’s about it.‬ I just- the reminders help. So, I get those reminders every week and just the fact that it doesn’t take long to fill out the symptoms. I like that it’s an easy pick on the scale and you don’t have to necessarily put in verbiage if you don’t want to. But there’s an opportunity, I think at the end to do that.”‬ ‬‬‬‬‬‬‬‬‬‬‬‬‬‬‬‬‬‬‬‬‬‬(Participant 16)‬‬‬‬‬‬‬‬‬‬‬‬‬
	Low	“So, I honestly did not use it as often as I thought that I would. But from what I recall- I’ve used it, it’s been a few months since I’ve recorded anything, but it was user friendly and pretty straightforward from what I recall.‬ ‬‬‬‬‬‬‬‬‬‬‬‬‬‬‬‬‬‬‬‬‬‬‬‬‬‬‬‬‬‬‬‬‬‬‬‬‬‬‬‬‬‬‬‬‬‬‬‬‬‬‬‬‬‬‬‬‬‬‬‬‬‬‬‬‬‬‬‬‬‬‬‬‬‬‬‬‬‬‬‬‬‬‬‬‬‬‬‬(Participant 28) “It, for me and my age, I find it difficult to read that much. The font is kind of small, so it makes it a little bit more difficult. Probably in hindsight, it would have been better to maybe watch- to have it on a tablet, an iPad, or something that is a bit bigger. But I don’t carry one of those around with me all the time.” (‬‬‬‬‬‬‬‬‬‬‬‬‬‬‬‬‬‬‬‬‬‬Participant 30)‬‬‬‬‬‬‬‬‬‬‬‬‬
Acceptability		
	High	“I like it. It’s very easy. It’s not a hindrance to me being out and about my day, anything like that.‬”‬‬‬‬‬‬‬‬‬‬‬‬‬‬‬‬‬‬‬‬‬‬‬ (Participant 4) I think it’s nice, concise, easy to use. It doesn’t take up much time.‬ It’s nice and easy to use. I like that you guys text me a reminder if I don’t remember to do it.‬ (Participant 8)
	Low	“So, I think for me, if I were to start having more symptoms, I probably would want to track it a little bit more. But because I’m not really having symptoms, I was kind of like, I don’t really need to go in and log anything, because I don’t really have anything to log.‬” (Participant 28) “Well, I didn’t use it. I didn’t have any what I would think of as extreme symptoms that I thought I needed a specialist for...So, why bother to go to an app when I can just call and get an answer pretty quickly? And if I need some kind of help, they can get it for me. And they did.” (Participant 29)
Introduction to palliative care (component 1)		
	High	“I think, like I said, the website was very simplistic. And I think the paper went a little bit more in depth. So that if you had questions, or didn’t quite understand something, or this was a completely new and foreign topic to you, you could look back at it and be able to get more questions answered on there.”‬ (Participant 8) “I think the things I learned from the reading, whether it was the website or the handout, I can’t remember. But, were just things I hadn’t thought about in addition to pain. I mean, I hadn’t really thought about things like appetite and constipation, depression and anxiety. I hadn’t really thought about those things as being something that palliative care would address. So, just seems to me there’s more to it than what I ever realized before.‬” (Participant 12)
	Low	“I really don’t remember. I kind of knew about palliative care, like I said, from my sister, and what she does for a living. But other than that, I don’t really remember the website.‬” (Participant 24) “We used that print out thing.‬ I really can’t tell you or explain it to‬ you. Yes, my sister explained it to me.‬ She explained what the piece of paper was telling us.‬” ‬‬‬‬‬‬‬‬‬‬‬‬‬‬‬‬‬‬‬‬‬‬‬‬‬‬‬‬‬‬‬‬‬‬‬‬‬‬‬‬‬‬‬‬‬‬‬‬‬‬‬‬‬‬‬‬‬‬‬‬‬‬‬‬‬‬(Participant 26)‬‬‬‬‬‬‬‬‬‬‬‬‬‬‬‬‬‬‬‬‬‬‬‬‬‬‬‬‬‬‬‬‬‬‬‬‬‬‬
Text reminders (component 2)		
	High	“I appreciate the text reminder every Monday.‬ Having a notification is great and having it at the same time, same day is also good.”‬ ‬‬‬‬‬‬‬‬‬‬‬‬‬‬‬‬‬‬‬‬‬‬(Participant 17)‬‬‬‬‬‬‬‬‬‬‬‬‬ “The text notification is perfect. As soon as I get it, I pin it to the top of my texts to remind myself to make sure I do it. So, no, that’s perfect.” (Participant 19)
	Low	“It was nice to have the reminders come. Oh, your survey is due, please answer it as soon as you can, or whatever. I didn’t feel pressured to do any of it, but. And like I said, I had very few symptoms, so there was really nothing to say.‬” (Participant 24) “I think mentally it was a little bit draining...I think when you’re going through it [cancer], it’s kind of nice to have a break from thinking about it. Maybe that’s a little bit why when it popped up as reminder, it was kind of like, ‘Oh, I don’t really want to think about this right now.’” (Participant 28)
Symptom reporting (component 3)		
	High	“It was pretty easy, I mean there wasn’t a lot of questions, so.‬” ‬‬‬‬‬‬‬‬‬‬‬‬‬‬‬‬(Participant 5) “I think giving me a scale from 0 to 10, it’s helpful for me to easily choose. And also display pain, no pain, worst possible pain. Let me understand how the scale works is making it very easy to select. So, I can just take click on number. And also, I can even skip this question if I prefer not to answer. That’s helpful.”‬ ‬‬‬‬‬‬‬‬(Participant 17)
	Low	“I used my cell phone. Yeah. I am pretty efficient with the computers and stuff, so. Using a cellphone or whatever to answer the questions is again, easy. And I didn’t have any issues with any of it.‬ Click and go. Click your answers down if there’s anything you need to spell out, you could. There’s plenty of area for that. Not just yes or no. And if you need to spell out something you could put down whatever symptoms you were having. And that was pretty well, easy to use‬.” ‬‬‬‬‬‬‬‬‬‬‬‬‬‬‬‬‬‬‬‬‬‬‬‬‬‬‬‬‬‬‬‬‬‬‬‬‬‬‬‬‬‬‬‬‬‬‬‬‬‬‬‬‬‬‬‬‬‬‬‬‬‬‬‬‬‬‬‬‬‬‬‬‬(Participant 24)‬‬‬‬‬‬‬‬‬‬‬‬‬ “Yes, I did use it. I know I did once, and I’m not sure if the second time it took or went through...And then afterwards, I just thought I’m not even doing this anymore.” (Participant 31)
Palliative care self-referral (component 4)		
	High	“I thought it was okay, but I haven’t had to request that yet.‬ Well, I like that the option is out there if I needed it. So, it’s just good to know that it’s there and in the background and available to me if I should need it.‬” ‬‬‬‬‬‬‬‬‬‬‬‬‬‬‬‬‬‬‬‬‬‬‬‬‬‬‬‬‬‬‬‬‬‬‬‬‬‬‬‬‬‬‬‬‬‬‬‬‬‬‬‬‬‬‬‬‬‬‬‬‬‬‬‬‬‬(Participant 11)‬‬‬‬‬‬‬‬‬‬‬‬‬ “Yeah, I think that’s good because I think it’s a very- to me it seems like a personal choice that somebody has to make. And I do think that you never know when somebody might get to that position that they feel like they need that extra assistance. So, having the access to do that. I mean, some people might feel like they have to wait until their next appointment or till the doctor suggests it. So, I think it’s nice to kind of, I don’t know, arm your patients with the tools that they need and know that they don’t have to go through their doctor or wait on an appointment but that’s something they can reach out to when they need it.”‬ ‬‬‬‬‬‬‬‬(Participant 16)
	Low	“Well, I supposed if I’d had more symptoms or something, I would have requested that, but. Like I said, I never even got so much as nauseous after my treatments and stuff. So, I really didn’t think I needed much palliative care.” (Participant 24) “I think that’s good. Did I request one? No.‬ Well, if I do, I would use OSU MyChart and send a message to palliative care.‬” ‬‬‬‬‬‬‬‬‬‬‬‬‬‬‬‬‬‬‬‬‬‬‬‬‬‬‬‬‬‬‬‬‬‬‬‬‬‬‬‬‬‬‬‬‬‬(Participant 26)‬‬‬‬‬‬‬‬‬‬‬‬‬
Oncology team callback (component 5)		
	High	“I have not because at least at this point in the trial, I’m down there every single week already anyway. So, I’m discussing symptoms when I’m there.‬ I think it’s great because, like I said, sometimes going through the regular channels or trying to figure out how do I log into the portal and message my doctor. If this is somebody that’s a brand-new patient, those things often can be daunting.‬” ‬‬‬‬‬‬‬‬‬‬‬‬‬‬‬‬‬‬‬‬‬‬‬‬‬‬‬‬‬‬‬‬‬‬‬‬‬‬‬‬‬‬‬‬‬‬‬‬‬‬‬‬‬‬‬‬‬‬‬‬‬‬‬‬‬‬‬‬‬‬(Participant 8)‬‬‬‬‬‬‬‬‬‬‬‬‬ “I think honestly, if something came up that I feel like I needed to reach out to my doctor, I would probably just call in at the office. That would be my first gut reaction.‬” ‬‬‬‬‬‬‬‬(Participant 16)
	Low	“Well, I think you should have it, but I think the patient should know that if they go to MyChart, they can direct contact the team.‬” (Participant 27) “I call. If I don’t call, well, I’ve had several times I needed information or something. And I every time I called...I went straight to the source. I called the doctor’s office.‬” (Participant 29)
Suggestions for program improvement		
	High	“I could give a section in there to where actually someone could document how they’re feeling in their own words. That might be helpful.”‬ ‬‬‬‬‬‬‬‬‬‬‬‬‬‬‬‬‬‬‬‬‬‬‬‬‬‬(Participant 2) “I don’t think with numbers you can fully explain it. I think it needs to be more patient-centric because everybody’s different and everybody reacts to it differently. The first time I went through chemo I didn’t feel anything. I really felt it this time. So, I think there’s - I think it needs to be a little more in depth.”‬ ‬‬‬‬‬‬‬‬‬‬‬‬‬‬‬‬‬‬(Participant 5) “It would be more beneficial if there was a way to say, have you experienced any new symptoms this week? And then like whatever you type in, then it’ll ask about that specific thing in the future.”‬ ‬‬‬‬‬‬(Participant 8) “Some of the symptoms it’s hard to quantify and I would forget the number I gave last time...So, if I can see what my last score is, it’s easy for me to make a judgment, right? Am I getting better or worse? So, I can move the score up or down from last time.‬ ‬‬‬‬(Participant 17)
	Low	“I guess the only thing that might help is to be able to explain why this is important. I got more of a feel of we’re just studying this app and seeing how it works for people and if you do it, fine, if you don’t do it, fine. That’s up to you but you’ll still be participating as long as you sign up or say you’ll do it or whatever. I guess I never got a real sense that it was really important.‬” ‬‬‬‬‬‬(Participant 29) “My recommendation would be to do a follow up reminder. Because it always seems like whatever time I got it, I was not in a position to be able to do it at that time. And then I could just completely forget about it. For then the next Monday I got one, or it was like the time I’m starting work or something whenever the reminder comes in. And so, it was just not a good time that I could take the time to do it at that- and then I just completely forgot about it.‬” (Participant 30)

^a^Table includes quotes from 17 participants.

### Participant Characteristics

All interview participants had a diagnosis of lung cancer and were followed by a thoracic medical oncologist, per eligibility requirements of the SyMPLER pilot study. Most interviewees were female (21/31, 67.7%), White (29/31, 93.5%), married (24/31, 77.4%), and had private health insurance (21/31, 67.7%; Table 1). Compared to the low-engagement group, participants in the high-engagement group tended to be younger (median age: 60 years vs 64 years, IQR 51-67 years vs IQR 58-70 years), have a college degree (8/21, 38.1% vs 1/10, 10% of group), live farther from the thoracic oncology clinic (median distance: 68 miles vs 19.4 miles, IQR 37.2-95.9 miles vs IQR 11.3-52.4 miles), and have metastatic disease at study enrollment (stage 4/extensive: 16/21, 76.2% vs 2/10, 20%). Twenty-six interviewees (83.9%) used the app at least once and were exposed to all components of the SyMPLER intervention. During interviews, 5 (16.1%) participants indicated they had self-referred to palliative care, and 4 (12.3%) noted they had requested a callback from the oncology team via the study app. A total of 2 patients self-referred to palliative care after completing their initial interview, and both agreed to complete a second interview about their experiences with palliative care self-referral through the app.

### SyMPLER Feasibility and Acceptability

Participants in both groups shared mostly positive perspectives about the SyMPLER intervention, stating that it was “user friendly,” “easy,” and “fast” (Table 2). Feasibility barriers differed by group, with high-engagement participants forgetting to report symptoms through the app (“I don’t always remember to use it”; Participant 7), while low-engagement participants cited low symptom burden (“don’t really have anything to log”; Participant 28), technology concerns (“I am electronically difficult”; Participant 23), and time constraints (“we just didn’t have time”; Participant 22). Three months after enrolling in the SyMPLER study, one participant requested help from the research staff to teach him how to navigate the app, which improved his ability to report symptoms:

I thought it was excellent. The only problem I had, which was my fault, not knowing until I learned, not knowing how to get in there and do the thing, fill out the questionnaires and all that stuff. That was my fault, not SyMPLER’s.‬

Participant 27, low engagement

### Impressions of SyMPLER Components

Participants shared their impressions about each component of SyMPLER (Table 2). Participants in both groups reported difficulty remembering details of the online and printed educational materials (component 1) that introduced palliative care upon study enrollment (“I don’t even know what that is. Palliative care.”; Participant 20, high engagement). Some participants in the high-engagement group reported they understood palliative care better after viewing the educational materials (“it completely changed my view”; Participant 17), with some reflecting on their prior exposures to palliative care (“I had several family members that were in it”; Participant 11). While most participants in the low-engagement group had little or no recollection of the educational materials, one participant reported strong emotions after viewing the website about palliative care:

I think that was pretty emotional for both of us...really the only thing kind of we knew about palliative care was more like end-of-life type care is sort of what we had in mind and so, it was a little bit, I don't know. It was a lot to watch the video, to be honest, because it was kind of like preparing for kind of a worst-case scenario is how it felt in moment.

Participant 28, low engagement

Participants in both groups reported overall positive opinions about the weekly text reminders (component 2), with some participants suggesting customization of the frequency, modality (eg, push notifications), and day/time when reminders were sent. Participants who reported symptoms through the study app (component 3) stated that the symptom survey (ESAS-r-CS [23]) was “simple,” “convenient,” and “pretty straight-forward.” Low-engagement participants cited technology-related concerns (“not being good at touching letters on a cell phone”; Participant 26) as a limitation to completing the ESAS-r-CS questionnaire via an app interface.

Participants endorsed the palliative care self-referral (component 4) as “a real good option,” although only 5 interviewees (3 high engagement; 2 low engagement) responded “yes” to this prompt. Participants in both groups cited tolerable symptom burden (“my symptoms are pretty much the normal for chemo...so, I’ve never had to use it”; Participant 7, high engagement), insufficient knowledge of palliative care (“I always put no because I didn’t understand what it was”; Participant 15, high engagement), and equivalency with hospice care (“more like end-of-life type care is sort of what we had in mind”; Participant 28, low engagement) as reasons for declining a palliative care referral. Participants indicated that palliative care may be beneficial for “other people,” while appreciating direct access via self-referral (“it wasn’t necessarily for me...but I can see the need for it and I thought it was good”; Participant 4, high engagement). One participant described how SyMPLER facilitated her ability to connect with palliative care services:

So, all along it was like, oh, there isn't really anything bad enough about what I'm going through to require this. But then I ended up having another surgery, and things went badly wrong and to the point where, I'm in a wheelchair now, and having a lot of pain from that. And that's when I asked for the palliative specialist...I mean, it made it very easy. Honestly, if I didn't have that on the app, I'm not sure I would have made the phone call. So, that was great.‬

Participant 12, high engagement, second interview

Participants also noted acceptability of the option to request a callback from their oncology team to discuss symptoms (“real helpful”), although only 4 participants used this option. Several participants voiced a preference for contacting their oncology team via telephone or patient portal (eg, MyChart) connected to the EHR.

### Suggestions for Program Improvement

Participants in both groups provided feedback on how to improve SyMPLER (Table 2). One participant asked for a “refresher” on the educational materials about palliative care. Participants suggested ways to make SyMPLER “more patient-centric,” such as adding additional free-text fields where patients could describe symptoms “in their own words.” Participants also expressed a desire to view reports or trends of past symptom scores (ESAS-r-CS [23]) to enable them to choose a score based on whether a symptom was better/worse than the previous day/week. To better integrate symptom reporting into their daily lives, participants requested further customization of the frequency (eg, twice weekly or biweekly) and timing (eg, day of the week or time of day) of text reminders delivered to their smartphones. Some participants also suggested that a follow-up reminder could be delivered a few days after the weekly text if they forgot to report symptoms in the app.

Most participants stated that they would recommend SyMPLER to other patients, although their reasons for recommending the program differed by group. While participants in the low-engagement group stated that they would recommend SyMPLER for the purpose of advancing cancer research (“But any help...we could provide for research purposes, I think is, can only be beneficial”; Participant 30), highly engaged participants identified how the program could improve patient care:

Well, it gets them connected to the right people when they need help. And I think sometimes with the science part of it, you can slip through it, kind of slip through the cracks a little bit.‬

Participant 2, high engagement

## Discussion

### Principal Findings

In this qualitative study, patients living with lung cancer identified key factors impacting their ability and willingness to use SyMPLER to report and seek help for cancer-related symptoms. By incorporating palliative care self-referral into an SMP, SyMPLER streamlines access to specialty palliative care by allowing patients to initiate a referral at their convenience, rather than waiting for their oncology teams to recognize the presence of uncontrolled symptoms and make a referral. While participants perceived that SyMPLER was helpful for providing direct access to palliative care, they also voiced uncertainty about when and why to request help from a palliative care specialist, thereby limiting use of the self-referral option. Key modifications to the intervention may enhance patient engagement with remote symptom reporting and increase their willingness to seek help for cancer symptom management.

Findings from this study should be considered in the context of symptom management theory, which describes the bidirectional interplay of an individual’s symptom experience (eg, symptom reporting) with symptom management (eg, palliative care referral and oncology team callback), ultimately impacting health outcomes such as HRQOL [9,10]. The relationship between symptom experience and management is an ideal target for intervention development, as multiple studies have demonstrated gaps in patient-clinician communication about cancer symptoms, frequently underestimating patients’ needs for symptom evaluation and treatment [7,8]. For this reason, SyMPLER aims to augment the symptom experience-management linkage by providing patients with an easy-to-use digital interface to report symptoms followed by streamlined options to seek help for uncontrolled symptoms when needed. Through purposive sampling based on level of engagement with the app, this study uniquely compares the perceptions of patients who were highly engaged in symptom reporting with those of patients who had limited use of the SMP. Between-group differences reflect how the 3 domains of symptom management theory—person, disease, and environment—influence participants’ perspectives of how SyMPLER affected their symptom experience, management, and health outcomes during cancer care [9].

Person-level variables (eg, age and education) are intrinsic to how an individual perceives and responds to the symptom experience, management options, and health changes [9]. Person-level variables play an important role in the success of any digital health intervention that relies on the patient’s ability and willingness to repeatedly engage with the technology. While nearly all patients screened for SyMPLER had smartphone access, low-engagement interviewees cited technological barriers affecting their ability to report symptoms via the study app. While smartphone ownership is associated with higher use of internet-based resources [41], disparities in digital health literacy continue to persist even among smartphone users, particularly affecting older adults [42] and individuals with lower income and/or education [43]. To address this digital divide, patients may benefit from educational outreach delivered by nursing staff or lay navigators to increase their understanding and engagement with symptom monitoring outside the health care setting [44]. The benefit of brief outreach was noted by the low-engagement participant who began reporting symptoms after asking study staff to show him how to navigate the study app. While this qualitative study was not powered to detect between-group differences in participant demographics, high-engagement interviewees tended to be younger, with a higher proportion having a college degree compared with low-engagement interviewees. Differences in person-level variables between high-engagement and low-engagement participants, if detected in the larger SyMPLER pilot study, could provide valuable information on which patients may benefit from brief educational outreach to improve engagement with symptom reporting.

Disease-level variables (eg, diagnosis and symptom burden) influence the duration, fluctuation, and trajectory of the symptom experience as well as the selection of management options that can most effectively improve symptom-related outcomes. In lung cancer, higher stage disease portends higher symptom burden [3]. This is reflected in low-engagement participants, a much lower proportion of whom had metastatic disease compared to the high-engagement group, reporting low symptom burden as a barrier to using SyMPLER for symptom reporting. While several studies have described clinical decline from metastatic disease as a barrier to completing symptom surveys [15,21], other studies report low symptom burden as a barrier to SMP engagement [45,46]. Findings from this qualitative study add credence to the paradox that patients living with cancer must feel well enough, yet not too well, to engage with an SMP. Participants in this study offered suggestions, such as the ability to view past survey responses and visualize trends in symptom scores, as potential strategies to overcome this paradox and improve long-term engagement with an SMP.

Most participants across both groups shared positive views of weekly text messages—a type of environmental cue—reminding them to report symptoms via SyMPLER. Reminders, typically delivered via SMS text or push notification to a smartphone device, are instrumental in promoting patient engagement with remote symptom monitoring [15]. Beyond SMPs, text reminders increase patient completion of health-related tasks, including cancer screenings [47], vaccinations [48], and medical appointments [49]. In SyMPLER, interviewees noted acceptance of text reminders, suggesting the cues were helpful in reminding them to complete a potentially ignorable task (eg, logging symptoms). In addition, participants described the need to customize the day and time at which text reminders are delivered to better integrate symptom reporting with the competing demands of patients’ daily lives (eg, returning to work [50]). Participants also suggested follow-up reminders, delivered when a task remains incomplete, as another strategy to encourage habitual symptom reporting in future iterations of SyMPLER.

Regarding palliative care self-referral, participants’ comments suggested a more complex picture framed by person, disease, and environmental domains. This complexity is particularly evident as most participants voiced support for palliative care self-referral while few actually used it; this may demonstrate a disconnect between perceived acceptability and real-world uptake. First, person-level variables (eg, education level) may have contributed to a participant’s ability to recall details about the educational materials introducing palliative care at study enrollment. While a single viewing of online educational materials may improve patients’ knowledge of and receptivity to palliative care [51], education alone is not enough to empower most patients to request palliative care for symptom management [52].

Second, while disease-level variables (eg, cancer stage) often drive symptom burden [3], participants were prompted to consider palliative care self-referral each time they reported symptoms via SyMPLER, regardless of symptom severity. While this enabled participants to request palliative care at any time and for any reason, participants voiced uncertainty about when or why to self-refer—an internal struggle also reported by clinicians when deciding whether to refer patients to specialty palliative care [53]. Future addition of symptom-triggered prompts when survey scores increase or exceed predefined thresholds, accompanied by “just-in-time” education about palliative care, may better inform patients when to seek help from palliative care for symptom management.

Finally, findings from this study showed how environmental variables, such as exposure to palliative and/or hospice care used by family and friends, framed participants’ perspectives about palliative care self-referral. While most participants across groups reported acceptance of SyMPLER’s palliative care self-referral option, several described palliative care as a service needed for “other people,” with 1 participant feeling emotionally triggered by the idea of palliative care, equating it to end-of-life care. These perspectives highlight the need for ongoing outreach efforts to normalize the concept of and educate patients about palliative care as an evidence-based intervention known to improve symptoms and HRQOL concurrent with cancer care [19].

This study has several limitations. First, we only interviewed participants exposed to the SyMPLER intervention. We may have obtained more diverse perspectives on remote symptom monitoring, particularly regarding technological barriers, if we had interviewed patients who declined SyMPLER enrollment due to technological concerns or lack of interest. While smartphone access was not a limitation among patients screened for SyMPLER, digital literacy may have contributed to app engagement, but this was not measured. Second, few interviewees opted for a palliative care self-referral, which limited our ability to ascertain why patients seek palliative care and whether these reasons fit within the current referral criteria recommended by consensus guidelines. As this was a qualitative study, no survey measures of symptom burden were collected at the time of interviews, although several participants reflected on how their symptoms, or lack thereof, contributed to their use of SyMPLER. Third, SyMPLER was piloted only among patients with lung cancer, which suggests opportunities to consider the impacts in other cancer populations. Although our study population lacks racial and ethnic diversity, it is well represented across education levels, insurance payer sources, and residential proximity to the cancer center. Females are overrepresented, which echoes a higher proportion of women enrolled in the SyMPLER pilot study. Finally, participant characteristics in each group differed by age, education, and cancer stage, which may have influenced patients’ engagement with SyMPLER, independent of their symptom burden.

### Conclusion

In this qualitative study, participants offered rich perspectives on the feasibility and acceptability of SyMPLER that differed by their level of engagement with the digital SMP. Most participants noted acceptance of the option to self-refer to palliative care, although feasibility was limited by low uptake of palliative care self-referral. Like clinicians, patients struggle with when and why to consult with a palliative care specialist, suggesting a need for stronger guidance on the optimal timing for palliative care consultation during a patient’s disease course.

## Appendix

Multimedia Appendix 1 Annotated Symptom Monitoring Program Linked to Electronic Referrals (SyMPLER) qualitative interview guide.

Multimedia Appendix 2 Symptom Monitoring Program Linked to Electronic Referrals (SyMPLER) qualitative interview codebook.

Multimedia Appendix 3 SRQR checklist.

## Abbreviations

CAPC: Center to Advance Palliative Care
EHR: electronic health record
ESAS: Edmonton Symptom Assessment System
ESAS-r-CS: Edmonton Symptom Assessment System–Revised Version Including Constipation and Sleep
HRQoL: health-related quality of life
IRB: Institutional Review Board
mHealth: mobile health
QoL: quality of life
SMP: symptom monitoring program
SRQR: Standards for Reporting Qualitative Research
SyMPLER: Symptom Monitoring Program Linked to Electronic Referrals

## Data Availability

The datasets generated and analyzed during this study are not publicly available due to potentially identifiable patient information but are available from the corresponding author on reasonable request under a data sharing agreement.

## References

[1] Radbruch L, De Lima L, Knaul F, Wenk R, Ali Z, Bhatnaghar S, Blanchard C, Bruera E, Buitrago R, Burla C, Callaway M, Munyoro EC (2020). Redefining palliative care—a new consensus-based definition. J Pain Symptom Manage.

[2] Ferrell BR, Twaddle ML, Melnick A, Meier DE (2018). National Consensus Project Clinical Practice Guidelines for Quality Palliative Care Guidelines, 4th Edition. J Palliat Med.

[3] Walling AM, Weeks JC, Kahn KL, Tisnado D, Keating NL, Dy SM, Arora NK, Mack JW, Pantoja PM, Malin JL (2015). Symptom prevalence in lung and colorectal cancer patients. J Pain Symptom Manage.

[4] Temel JS, Greer JA, Muzikansky A, Gallagher ER, Admane S, Jackson VA, Dahlin CM, Blinderman CD, Jacobsen J, Pirl WF, Billings JA, Lynch TJ (2010). Early palliative care for patients with metastatic non-small-cell lung cancer. N Engl J Med.

[5] Temel JS, Greer JA, El-Jawahri A, Pirl WF, Park ER, Jackson VA, Back AL, Kamdar M, Jacobsen J, Chittenden EH, Rinaldi SP, Gallagher ER, Eusebio JR, Li Z, Muzikansky A, Ryan DP (2017). Effects of early integrated palliative care in patients with lung and GI cancer: a randomized clinical trial. J Clin Oncol.

[6] Chandwani KD, Zhao F, Morrow GR, Deshields TL, Minasian LM, Manola J, Fisch MJ (2017). Lack of patient-clinician concordance in cancer patients: its relation with patient variables. J Pain Symptom Manage.

[7] Newell S, Sanson-Fisher RW, Girgis A, Bonaventura A (1998). How well do medical oncologists' perceptions reflect their patients' reported physical and psychosocial problems? Data from a survey of five oncologists. Cancer.

[8] Xiao C, Polomano R, Bruner DW (2013). Comparison between patient-reported and clinician-observed symptoms in oncology. Cancer Nurs.

[9] Dodd M, Janson S, Facione N, Faucett J, Froelicher ES, Humphreys J, Lee K, Miaskowski C, Puntillo K, Rankin S, Taylor D (2001). Advancing the science of symptom management. J Adv Nurs.

[10] Carrasco S (2021). Patients' communication preferences around cancer symptom reporting during cancer treatment: a phenomenological study. J Adv Pract Oncol.

[11] Andikyan V, Rezk Y, Einstein MH, Gualtiere G, Leitao MM, Sonoda Y, Abu-Rustum NR, Barakat RR, Basch EM, Chi DS (2012). A prospective study of the feasibility and acceptability of a Web-based, electronic patient-reported outcome system in assessing patient recovery after major gynecologic cancer surgery. Gynecol Oncol.

[12] Basch E, Deal AM, Kris MG, Scher HI, Hudis CA, Sabbatini P, Rogak L, Bennett AV, Dueck AC, Atkinson TM, Chou JF (2016). Symptom monitoring with patient-reported outcomes during routine cancer treatment: a randomized controlled trial. J Clin Oncol.

[13] Basch E, Deal AM, Dueck AC, Scher HI, Kris MG, Hudis C, Schrag D (2017). Overall survival results of a trial assessing patient-reported outcomes for symptom monitoring during routine cancer treatment. JAMA.

[14] Basch E, Schrag D, Henson S, Jansen J, Ginos B, Stover AM, Carr P, Spears PA, Jonsson M, Deal AM, Bennett AV, Thanarajasingam G (2022). Effect of electronic symptom monitoring on patient-reported outcomes among patients with metastatic cancer: a randomized clinical trial. JAMA.

[15] Xia Y, Guan X, Zhu W, Wang Y, Shi Z, He P (2025). Effectiveness of symptom monitoring on electronic patient-reported outcomes (ePROs) among patients with lung cancer: a systematic review and meta-analysis. NPJ Digit Med.

[16] Rocque GB, Dent DN, Ingram SA, Caston NE, Thigpen HB, Lalor FR, Jamy OH, Giri S, Azuero A, Young Pierce J, McGowen CL, Daniel CL, Andrews CJ (2022). Adaptation of remote symptom monitoring using electronic patient-reported outcomes for implementation in real-world settings. JCO Oncol Pract.

[17] Kroenke K, Talib TL, Stump TE, Kean J, Haggstrom DA, DeChant P, Lake KR, Stout M, Monahan PO (2018). Incorporating PROMIS symptom measures into primary care practice—a randomized clinical trial. J Gen Intern Med.

[18] Swarm RY, Youngwerth JM, Agne JL, Anitescu M, Are M, Buga S, Butler T, Chwistek M, Cleary J, Copenhaver D (2025). Adult cancer pain, Version 2.2025, NCCN Clinical Practice Guidelines in Oncology. J Natl Compr Canc Netw.

[19] Sanders JJ, Temin S, Ghoshal A, Alesi ER, Ali ZV, Chauhan C, Cleary JF, Epstein AS, Firn JI, Jones JA, Litzow MR, Lundquist D (2024). Palliative care for patients with cancer: ASCO guideline update. J Clin Oncol.

[20] Kuo JC, Graham DM, Salvarrey A, Kassam F, Le LW, Shepherd FA, Burkes R, Hollen PJ, Gralla RJ, Leighl NB (2020). A randomized trial of the electronic Lung Cancer Symptom Scale for quality-of-life assessment in patients with advanced non-small-cell lung cancer. Curr Oncol.

[21] Parikh RB, Schriver E, Ferrell WJ, Wakim J, Williamson J, Khan N, Kopinsky M, Balachandran M, Gabriel PE, Schuchter LM, Patel MS, Shulman LN, Manz CR (2023). Remote patient-reported outcomes and activity monitoring to improve patient-clinician communication regarding symptoms and functional status: a randomized controlled trial. JCO Oncol Pract.

[22] Get Palliative Care.

[23] Hannon B, Dyck M, Pope A, Swami N, Banerjee S, Mak E, Bryson J, Rodin G, Ridley J, Lo C, Le LW, Zimmermann C (2015). Modified Edmonton Symptom Assessment System including constipation and sleep: validation in outpatients with cancer. J Pain Symptom Manage.

[24] Harris PA, Swafford J, Serdoz ES, Eidenmuller J, Delacqua G, Jagtap V, Taylor RJ, Gelbard A, Cheng AC, Duda SN (2022). MyCap: a flexible and configurable platform for mobilizing the participant voice. JAMIA Open.

[25] Ceronsky L, Goldstein N, Horton JR, Johnson K, Kang TI, Loengard A, Panke J, Popp B, Schisler R Palliative care patient education handouts. Get Palliative Care.

[26] Business text messaging. Twilio.

[27] Caraceni A, Lo Dico S, Zecca E, Brunelli C, Bracchi P, Mariani L, Garassino MC, Vitali M (2020). Outpatient palliative care and thoracic medical oncology: referral criteria and clinical care pathways. Lung Cancer.

[28] Mazza GL, Dueck AC, Ginos B, Jansen J, Deal AM, Carr P, Blinder VS, Thanarajasingam G, Jonsson M, Lee MK (2024). Optimization of alert notifications in electronic patient-reported outcome (ePRO) remote symptom monitoring systems (AFT-39). Qual Life Res.

[29] Hui D, Heung Y, Bruera E (2022). Timely palliative care: personalizing the process of referral. Cancers (Basel).

[30] Agne JL, Bertino EM, Grogan M, Benedict J, Janse S, Naughton M, Eastep C, Callahan M, Presley CJ (2021). Too many appointments: assessing provider and nursing perception of barriers to referral for outpatient palliative care. Palliat Med Rep.

[31] Hassett MJ, Cronin C, Tsou TC, Wedge J, Bian J, Dizon DS, Hazard-Jenkins H, Osarogiagbon RU, Wong S, Basch E, Austin T, McCleary N, Schrag D (2022). eSyM: an electronic health record-integrated patient-reported outcomes-based cancer symptom management program used by six diverse health systems. JCO Clin Cancer Inform.

[32] Finney Rutten LJ, Ruddy KJ, Chlan LL, Griffin JM, Herrin J, Leppin AL, Pachman DR, Ridgeway JL, Rahman PA, Storlie CB (2020). Pragmatic cluster randomized trial to evaluate effectiveness and implementation of enhanced EHR-facilitated cancer symptom control (E2C2). Trials.

[33] Tenny S, Brannan J, Brannan G (2026). Qualitative Study. StatPearls.

[34] Saunders B, Sim J, Kingstone T, Baker S, Waterfield J, Bartlam B, Burroughs H, Jinks C (2018). Saturation in qualitative research: exploring its conceptualization and operationalization. Qual Quant.

[35] Savin-Baden M, Major CH (1999). Qualitative Research: The Essential Guide to Theory and Practice.

[36] Allemang B, Sitter K, Dimitropoulos G (2022). Pragmatism as a paradigm for patient-oriented research. Health Expect.

[37] Braun V, Clarke V (2021). Thematic Analysis: A Practical Guide.

[38] Green J, Thorogood N (2018). Qualitative Methods for Health Research.

[39] Guest G, Bunce A, Johnson L (2006). How many interviews are enough? An experiment with data saturation and variability. Field Methods.

[40] O'Brien BC, Harris IB, Beckman TJ, Reed DA, Cook DA (2014). Standards for reporting qualitative research: a synthesis of recommendations. Acad Med.

[41] Oshima SM, Tait SD, Thomas SM, Fayanju OM, Ingraham K, Barrett NJ, Hwang ES (2021). Association of smartphone ownership and internet use with markers of health literacy and access: cross-sectional survey study of perspectives from Project PLACE (Population Level Approaches to Cancer Elimination). J Med Internet Res.

[42] Leader AE, Capparella LM, Waldman LB, Cammy RB, Petok AR, Dean R, Shimada A, Yocavitch L, Rising KL, Garber GD, Worster B, Dicker AP (2021). Digital literacy at an urban cancer center: implications for technology use and vulnerable patients. JCO Clin Cancer Inform.

[43] Chisolm RB, Tomori C, Spaulding EM (2026). The impact of digital health literacy on patient adherence, self-management, and follow-up care: an integrative review. Telemed J E Health.

[44] McAlearney AS, Walker DM, Sieck CJ, Fareed N, MacEwan SR, Hefner JL, Di Tosto G, Gaughan A, Sova LN, Rush LJ, Moffatt-Bruce S, Rizer MK, Huerta TR (2022). Effect of in-person vs video training and access to all functions vs a limited subset of functions on portal use among inpatients: a randomized clinical trial. JAMA Netw Open.

[45] Bhargava R, Keating B, Isenberg SR, Subramaniam S, Wegier P, Chasen M (2021). RELIEF: a digital health tool for the remote self-reporting of symptoms in patients with cancer to address palliative care needs and minimize emergency department visits. Curr Oncol.

[46] Nielsen AS, Kidholm K, Kayser L (2020). Patients' reasons for non-use of digital patient-reported outcome concepts: a scoping review. Health Informatics J.

[47] Huf S, Kerrison RS, King D, Chadborn T, Richmond A, Cunningham D, Friedman E, Shukla H, Tseng F, Judah G, Darzi A, Vlaev I (2020). Behavioral economics informed message content in text message reminders to improve cervical screening participation: two pragmatic randomized controlled trials. Prev Med.

[48] Milkman KL, Gandhi L, Patel MS, Graci HN, Gromet DM, Ho H, Kay JS, Lee TW, Rothschild J, Bogard JE (2022). A 680,000-person megastudy of nudges to encourage vaccination in pharmacies. Proc Natl Acad Sci U S A.

[49] Werner K, Alsuhaibani SA, Alsukait RF, Alshehri R, Herbst CH, Alhajji M, Lin TK (2023). Behavioural economic interventions to reduce health care appointment non-attendance: a systematic review and meta-analysis. BMC Health Serv Res.

[50] Butow P, Laidsaar-Powell R, Konings S, Lim CYS, Koczwara B (2020). Return to work after a cancer diagnosis: a meta-review of reviews and a meta-synthesis of recent qualitative studies. J Cancer Surviv.

[51] McLouth LE, Borger T, Bursac V, Hoerger M, McFarlin J, Shelton S, Shelton B, Shearer A, Kiviniemi MT, Stapleton JL, Mullett T (2023). Palliative care use and utilization determinants among patients treated for advanced stage lung cancer care in the community and academic medical setting. Support Care Cancer.

[52] McLouth LE, Stapleton JL, Bursac V, Zelaya CM, Shelton BJ, Thakur K, Hands I, Blu C, Chih M, McFarlin JM (2024). Piloting a patient tool to aid palliative care referrals during advanced lung cancer treatment. J Pain Symptom Manage.

[53] Enguidanos S, Cardenas V, Wenceslao M, Hoe D, Mejia K, Lomeli S, Rahman A (2021). Health care provider barriers to patient referral to palliative care. Am J Hosp Palliat Care.

