# Supplementary material for: Patient Experiences With a Symptom Monitoring and Self-Referral Program for Specialty Palliative Care After Lung Cancer Diagnosis: Qualitative Study
Source: JMIR Form Res. 2026 Sep 24;10:e95948. doi: 10.2196/95948 (PMC13650458; doi:10.2196/95948)
Supplement: Supplementary file 1 [file formative_v10i1e95948_app1.docx]

**Appendix 1: Annotated SyMPLER Qualitative Interview Guide**

***Annotation Legend***

|  | **Name [abbreviation]** | **Description** |
| --- | --- | --- |
| **Topic** | Feasibility [F]  Acceptability [A]  Component 1 [C1]  Component 2 [C2]  Component 3 [C3]  Component 4 [C4]  Component 5 [C5] | Ability to participate in the SyMPLER intervention  Willingness to participate in the SyMPLER intervention  Introduction to palliative care  Weekly SMS text reminders  On-demand symptom reporting via study app  Prompted option for palliative care self-referral  Prompted option for oncology team contact |
| **Domain^1^** | Person [P]  Disease [D]  Environment [E] | An individual’s perception and response to symptoms  An illness that causes or influences symptoms  Physical, social, and cultural contexts |
| **Dimension^1^** | Symptom Experience [SE]  Symptom Management [SM]  Outcomes [O] | Recognition and reporting of symptoms  Evaluation and response to treatment options  Health-related changes impacted by symptoms |

^1^ Domains and dimensions derived from the model of Symptom Management Theory

**Annotated Interview Guide**

***[Introduction]*** Thank you for speaking with me today. The purpose of this interview is to learn about your experience participating in the symptom monitoring program. As we talked about during the consent process, your participation is voluntary, and you do not need to answer any questions that you do not want to. You can also end the interview at any time without penalty or loss of any benefits to which you are otherwise entitled. Everything you tell us will be confidential. We will never use your name in any reports or articles. I would also like to record our session for the purposes of data collection for our research. The recording will not be used to identify you in any way. Do you have any questions before we start?

***[Section 1: Symptom Monitoring Program]*** To start, I would like to ask a few questions about what you think of the symptom monitoring program.

What is your overall opinion of the symptom monitoring program? [F, P]

What has been going well with the program? [F, P]

What has not gone well with the program? [F, P]

What was your experience like when completing the surveys on your cell phone or personal electronic device? [F, C3, P, SE]

What made it hard or difficult to complete these surveys? [F, C3, P, SE]

What made it easy to complete these surveys? [F, C3, P, SE]

How often did you receive notifications to complete surveys? [F, C2, E]

What made it hard or difficult to remember to complete these surveys? [F, C2, E]

What made it easy to remember to complete these surveys? [F, C2, E]

How well did the survey allow you to report or explain how you were feeling? [F, C2, P, SE]

Is there anything you would change about this program? [F, A, P]

Would you recommend this program to other patients with a cancer diagnosis? [A, P]

Why/Why not? (please explain) [A, P]

***[Section 2: Introduction to Palliative Care]*** When you first enrolled in the symptom monitoring program, you were asked to view a website about palliative care [C1].

What did you know about palliative care before viewing the website? [P, E]

What do you remember about the palliative care website? [F, P]

What did you learn about palliative care after viewing the website? [F, P]

What would you change about the website? [F, A, P]

Would you recommend the website to another patient wanting information on palliative care? [A, P]

You were also given a printed handout about palliative care. [C1]

Do you remember receiving the handout? [F, P, E]

If so, do you remember reading it? [F, P, E]

What did you learn about palliative care from the handout? [F, P]

What would you change about the handout? [F, A, P]

After reading the handout, do you think you knew enough about palliative care to request this service? [F, P]

What additional information would you have wanted to know about palliative care? [F, P]

Would you recommend the printed handout to another patient wanting information on palliative care? [A, P]

If you had to choose, did you prefer the website or the printed handout for the information provided? [A, P]

…If you thought both provided enough information you can say both equally.

***[Section 3: Palliative Care Self-Referral]*** After reporting symptoms through the weekly surveys, you were asked if you wanted to see a palliative care specialist during your next oncology visit. [C4]

What did you think about the option to request to see a palliative care specialist? [A, P, SM]

At any point, did you select “Yes” and request to see a palliative care specialist? [P, SM]

**[YES]:**

What made you decide to request palliative care? [P, D, SE, SM]

What was your experience like scheduling this appointment with a palliative care specialist? [F, P, E, SM]

What made it hard or difficult to schedule this appointment? [F, P, E, SM]

What made it easy to schedule this appointment? [F, P, E, SM]

How has palliative care affected your medical care? [P, O]

**[NO]:**

Do you think you might request palliative care in the future? Why or why not? [A, P, SE, SM]

What do you think about being able to request palliative care yourself? [A, P, E, SM]

***[Section 4: Oncology Team Contact]*** After reporting symptoms through the weekly surveys, you were also asked if you wanted a member of the medical team to contact you within 3 days to discuss your symptoms. [C5]

At any point, did you select “Yes” to request contact from the medical team? [P, SM]

**[YES]:**

Why did you want to be contacted by the medical team? [P, D, SE, SM]

Did the medical team contact you? [F, SM]

If so, how helpful was it to be contacted by the medical team? [A, P, SM, O]

**[NO]** Do you think you might request that the medical team contact you in the future through this program? Why or why not? [F, A, P, SM]

What do you think about being able to directly request contact from the medical team? [A, P, SM, O]

***[Conclusion]*** Thank you for taking time to speak with me today. Your comments were very helpful. Is there anything that we haven’t discussed that you would like to share?
