# Supplementary material for: Patient Experiences With a Symptom Monitoring and Self-Referral Program for Specialty Palliative Care After Lung Cancer Diagnosis: Qualitative Study
Source: JMIR Form Res. 2026 Sep 24;10:e95948. doi: 10.2196/95948 (PMC13650458; doi:10.2196/95948)
Supplement: Supplementary file 2 [file formative_v10i1e95948_app2.docx]

**Appendix 2: Symptom Monitoring Program Linked to Electronic Referrals (SyMPLER)**

**Qualitative Interview Codebook**

**General Guidance on Coding**

1. Coders should read through the entire codebook prior to coding any transcripts.
2. Coders should read through the transcript before coding.
3. While coders should aim to apply a single code that best fits the meaning of a passage, multiple codes should be applied when a passage meaningfully describes multiple codes.

**Coding Dictionary**

| Code | Definition | *Examples* |
| --- | --- | --- |
| *Background | Age, education, etc. ‬ | **Collected per SyMPLER parent study protocol* |
| Chit Chat | Conversation not related to topics being covered in interview guide. | *Random conversation about pets, bad service, etc.* |
| Feasibility & Acceptability of the Symptom Monitoring Program (SMP) | Feasibility   - Positives- things they liked - Negatives- things they didn’t like - Barriers to participation   Acceptability   - Recommend to others- would recommend to other patients, why or why not | *“I like it.‬ It helps me keep track. My only problem is, is when I look at it, I just focus basically on that day, does that make sense?‬ It’s easy to use.”*‬‬‬‬‬‬  *“I just would definitely recommend it and recommend that people make use of the opportunities that it offers so.‬”* |
| Suggestions for Improvement | Descriptions of   - Suggestions- things they would change | *“Really well. I could give a section in there to where actually someone could document how they're feeling in their own words. That might be helpful.‬”*‬‬ |
| Weekly Text Reminders | Descriptions of   - Positives- things they liked - Negatives- things they didn’t like - Suggestions- what they would change | *“I appreciate the text reminder every Monday.‬ Having a notification is great and having it at the same time, same day is also good.”‬*‬  *‬‬‬‬‬‬‬‬‬‬‬*  *“My recommendation would be to do a follow up reminder. Because it always seems like whatever time I got it, I was not in a position to be able to do it at that time. And then I could just completely forgot about it.”* |
| Symptom Reporting Surveys | Descriptions of   - Easy- what made them easy - Hard- what made them hard - Suggestions- what they would change | *“Just the nausea question. The majority of time, I don't have nausea, or if I do, I mark it. I always put 1 or 2, because I am not constantly nauseous. But it happens on occasion, kind of thing.‬”*‬‬‬  *“*W*hen it asks to schedule an appointment with palliative care, I never know because it’s not bad enough where I’m like, I need something. So, I never really know how to answer that question either. I always just put, no, and that I want to speak to my oncologist first.‬”*‬‬‬ |
| Education about Palliative Care | Descriptions of   - Background knowledge about palliative care (before viewing website) | *“Not really a whole lot.‬”*‬‬‬  *“Oh, I've known about it for a long time. For that I'm in healthcare, so palliative care can be helpful, and a lot of people deem it as end-of-life, but really, it's not. It's just to get you the right help you need when you need it, and that- yep.‬”*‬‬ |
| Palliative Care Website | - Impressions of palliative care website   - What was learned   - Suggestions for improvement   - Recommend to others- would recommend to other patients, why or why not | *“At this point in time, I don’t remember even the website.‬”*‬‬‬ |
| Palliative Care Printed Handout | - Impressions of palliative care printed handout   - What was learned   - Suggestions for improvement   - Recommend to others- would recommend to other patients, why or why not | *“So, I remember getting it, but I don’t remember now what was in it.‬”*‬‬  *“I remember reading it, but it’s been a minute.‬ I mean just what it consists of. But I mean, I didn't learn anything new. I already knew most of the stuff about palliative care.‬”*‬‬‬‬  ‬ |
| Palliative Care Self-Referral | Descriptions of   - Thoughts on option to request to see palliative care specialist (PCS) - Experience if requested to see PCS = yes - If PCS = no, why? | *“I think that would be a good thing. I still think people should talk to their oncologist first, and get their interpretation of what's going on and- but I guess if you really need it, then it's a good thing. I like having the option of being able to say okay, this is what's happening now, I need help. And being able to reach out to them and being able to get an appointment and see them and work things out.‬”*‬‬‬‬‬‬  *“I thought that was a real good option, although I never requested. But I just thought that was a real good option.‬”*‬‬ |
| Medical Team Contact | Descriptions of   - Thoughts on option to request to see medical team - Experience if requested = yes - If = no, why? | *“I think when I was going through that esophagitis, maybe I did. I am not 100 percent. But I think that’s a good thing also.‬”*‬‬‬  *“Yes, I did, and it wasn't within a few days. It was normally within an hour. So, that was real helpful.‬”*‬‬ |
| Z-hold | Things that seem important but don’t fit in existing code‬.‬‬‬‬‬‬‬ | **Questions not from interview guide* |
