# Supplementary material for: Patient Experiences With a Symptom Monitoring and Self-Referral Program for Specialty Palliative Care After Lung Cancer Diagnosis: Qualitative Study
Source: JMIR Form Res. 2026 Sep 24;10:e95948. doi: 10.2196/95948 (PMC13650458; doi:10.2196/95948)
Supplement: Supplementary file 3 [file formative_v10i1e95948_app3.docx]

**Appendix 3: Standards for reporting qualitative research (SRQR) checklist** [1]

Title: SyMPLER Palliative Care: a qualitative study of patient experiences with a symptom monitoring program and self-referral to specialty palliative care via a mobile health application

Authors: Julia L Agne MD^1,2^, Alice A Gaughan MS^3^, Laura J Rush DVM PhD^3^, Jennifer L Eramo MSN RN^3^, Anneliese Sinclair BA^3^, Madison M Grogan MPH^4^, Jessica S Merlin MD PhD MBA^1,2^, Jack Stevens PhD^5,6^, Robert Cronin MD MS MEng^7^, Tasleem J Padamsee PhD^8^, Carolyn J Presley MD MHS^4^, Ann Scheck McAlearney ScD MS^3,9^

1. Section of Palliative and Pain Research (SPARC), Division of Palliative Medicine, The Ohio State University Wexner Medical Center, Columbus, Ohio, USA
2. Center for Research Excellence in Supportive Care (CREST), The Ohio State University Comprehensive Cancer Center, Division of Medical Oncology, Columbus, Ohio, USA
3. The Center for the Advancement of Team Science, Analytics, and Systems Thinking in Health Services and Implementation Science Research (CATALYST), The Ohio State University College of Medicine, Columbus, Ohio, USA
4. Division of Medical Oncology, The Ohio State University Comprehensive Cancer Center, Columbus, Ohio, USA
5. Nationwide Children’s Hospital, Columbus, Ohio, USA
6. The Ohio State University Department of Pediatrics, Columbus, Ohio, USA
7. Department of Internal Medicine, The Ohio State University Wexner Medical Center, Columbus, Ohio, USA
8. Division of Health Services Management and Policy, College of Public Health, The Ohio State University, Columbus, Ohio, USA
9. The Ohio State University Department of Family and Community Medicine, Columbus, Ohio, USA

| No. | Topic | Items | Page no. |
| --- | --- | --- | --- |
| **Title and Abstract** | | | |
| S1 | Title | Concise description of the nature and topic of the study Identifying the study as qualitative or indicating the approach (e.g., ethnography, grounded theory) or data collection methods (e.g., interview, focus group) is recommended | Page 1 |
| S2 | Abstract | Summary of key elements of the study using the abstract format of the intended publication; typically includes background, purpose, methods, results, and conclusions | Page 2 |
| **Introduction** | | | |
| S3 | Problem formulation | Description and significance of the problem/phenomenon studied; review of relevant theory and empirical work; problem statement | Pages 3-5 |
| S4 | Purpose or research question | Purpose of the study and specific objectives or questions | Page 5 |
| **Methods** | | | |
| S5 | Qualitative approach and research paradigm | Qualitative approach (e.g., ethnography, grounded theory, case study, phenomenology, narrative research) and guiding theory if appropriate; identifying the research paradigm (e.g., postpositivist, constructivist/ interpretivist) is also recommended; rationale | Pages 8-10 |
| S6 | Researcher characteristics and reflexivity | Researchers’ characteristics that may influence the research, including personal attributes, qualifications/experience, relationship with participants, assumptions, and/or presuppositions; potential or actual interaction between researchers’ characteristics and the research questions, approach, methods, results, and/or transferability | Page 10 |
| S7 | Context | Setting/site and salient contextual factors; rationale | Pages 6-9 |
| S8 | Sampling strategy | How and why research participants, documents, or events were selected; criteria for deciding when no further sampling was necessary (e.g., sampling saturation); rationale | Pages 6-7 |
| S9 | Ethical issues pertaining to human subjects | Documentation of approval by an appropriate ethics review board and participant consent, or explanation for lack thereof; other confidentiality and data security issues | Page 6 |
| S10 | Data collection method | Types of data collected; details of data collection procedures including (as appropriate) start and stop dates of data collection and analysis, iterative process, triangulation of sources/methods, and modification of procedures in response to evolving study findings; rationale | Page 10 |
| S11 | Data collection instruments and technologies | Description of instruments (e.g., interview guides, questionnaires) and devices (e.g., audio recorders) used for data collection; if/how the instrument(s) changed over the course of the study | Page 10 |
| S12 | Units of study | Number and relevant characteristics of participants, documents, or events included in the study; level of participation (could be reported in results) | Page 11 |
| S13 | Data processing | Methods for processing data prior to and during analysis, including transcription, data entry, data management and security, verification of data integrity, data coding, and anonymization/de-identification of excerpts | Page 10 |
| S14 | Data analysis | Process by which inferences, themes, etc., were identified and developed, including the researchers involved in data analysis; usually references a specific paradigm or approach; rationale | Page 10 |
| S15 | Techniques to enhance trustworthiness | Techniques to enhance trustworthiness and credibility of data analysis (e.g., member checking, audit trail, triangulation); rationale | Page 10 |
| **Results/findings** | | | |
| S16 | Synthesis and interpretation | Main findings (e.g., interpretations, inferences, and themes); might include development of a theory or model, or integration with prior research or theory | Pages 11-15 |
| S17 | Links to empirical data | Evidence (e.g., quotes, field notes, text excerpts, photographs) to substantiate analytic findings | Pages 12-15 |
| **Discussion** | | | |
| S18 | Integration with prior work, implications, transferability, and contribution(s) to the field | Short summary of main findings; explanation of how findings and conclusions connect to, support, elaborate on, or challenge conclusions of earlier scholarship; discussion of scope of application/generalizability; identification of unique contribution(s) to scholarship in a discipline or field | Pages 15-18 |
| S19 | Limitations | Trustworthiness and limitations of findings | Page 18 |
| **Others** | | | |
| S20 | Conflict of interest | Potential sources of influence or perceived influence on study conduct and conclusions; how these were managed | Page 20 |
| S21 | Funding | Sources of funding and other support; role of funders in data collection, interpretation, and reporting | Page 20 |

Reference:

1.	O'Brien BC, Harris IB, Beckman TJ, Reed DA, Cook DA. Standards for reporting qualitative research: a synthesis of recommendations. Acad Med. 2014 Sep;89(9):1245–51. PMID: 24979285. doi: 10.1097/ACM.0000000000000388.
